# Microbial removal of nutrients from anaerobic digestate: assessing product-coupled and non-product-coupled approaches

**DOI:** 10.3389/fmicb.2023.1299402

**Published:** 2023-12-11

**Authors:** Eric Agyeman-Duah, Christopher C. Okonkwo, Victor C. Ujor

**Affiliations:** ^1^Fermentation Science and Metabolic Engineering Group, Department of Food Science, University of Wisconsin-Madison, Madison, WI, United States; ^2^Biotechnology Program, Department of Chemistry and Chemical Biology, The Roux Institute, Northeastern University, Portland, ME, United States

**Keywords:** anaerobic digestate, fumaric acid, phosphate-accumulating organisms, microbial nutrient removal, phosphate removal

## Abstract

Although anaerobic digestate contains >90% water, the high nutrient content of digestate makes it economically and technically intractable to treatment by existing wastewater treatment technologies. This study separately assessed the feasibility of nutrient removal from digestate by *Rhizopus delemar* DSM 905 and a culture of phosphate-accumulating organisms (PAOs). With *Rhizopus delemar* DSM 905, we investigated concomitant nutrient removal from digestate-supplemented medium and fumaric acid production, as a potentially economical strategy for digestate treatment. Following the cultivation of *R*. *delemar* DSM 905 in a fermentation medium containing 25% (v/v) digestate, the concentrations of Al, Cr, Cu, Fe, K, Mg, Mn, Pb, and Zn reduced 40, 12, 74, 96, 12, 26, 23%, ~18, and 28%, respectively. Similarly, the concentrations of total phosphorus, total nitrogen, phosphate (PO_4_-P), ammonium (NH_4_-N), nitrate (NO_3_-N), and sulfur decreased 93, 88, 97, 98, 69, and 13%, respectively. Concomitantly, cultures supplemented with 25 and 15% (v/v) digestate produced comparable titers of fumarate (~11 and ~ 17 g/L, respectively) to the digestate un-supplemented control cultures. With PAOs, we assessed the removal of total phosphorus, total nitrogen, PO_4_-P, and NH_4_-N, of which the concentrations reduced 86, 90%, ~99, and 100%, respectively in 60% (v/v) digestate. This study provides additional bases for microbial removal of excess nutrients from anaerobic digestate, with the potential to engender future water recovery from this waste stream that is currently largely recalcitrant to treatment.

## 1 Introduction

Although anaerobic digestion (AD) represents an excellent technology for treating organic wastes (Fagbohungbe et al., 2017; Kumar et al., 2022), the waste derived from AD [i.e., anaerobic digestate (ADE)] is increasingly becoming a burden on the AD industry and the environment. This is because, ADE produced by large AD facilities outstrip local fertilizer demands. Consequently, ADE is commonly stored in lagoons, a practice that poses considerable hazard to the environment. In addition to contributing to the emission of greenhouse gases (GHGs) due to continued release of methane and CO_2_ from lagoons in the event of flooding, ADE introduces substantial amounts of ammonium and phosphate into the environment, which engender eutrophication, an increasingly worsening environmental challenge (Paerl and Barnard, 2020; Ralston and Moore, 2020). Transporting heavy, water-replete ADE from where it is produced to where it may be needed as fertilizer is expensive, with the attendant emission of CO_2_. Depending on the AD feedstock, fresh ADE can contain over 3,000 and 5,000 mg/L ammonium and phosphate, respectively (Paerl and Barnard, 2020; Kumar et al., 2022), whereas municipal sewage contains about 40 and 20 mg/L, respectively. Consequently, ADE is technically and economically intractable to treatment by standard wastewater treatment technologies. Additionally, prolonged/heavy application of ADE on land as fertilizer exerts toxicological effects on soil microbes, which adversely affects vital nitrifying bacteria (Franchino et al., 2016; Bankston et al., 2020). ADE typically contains high levels of sodium. As a result, when extensively applied on agricultural land as fertilizer, ADE increases soil salinity and electrical conductivity thereby damaging the hydraulic properties of soil (Voelkner et al., 2015; Holatko et al., 2021). Similarly, prolonged or heavy application of ADE on arable land has been shown to increase soil hydrophobicity, which impairs seed germination (Wallach, 2010). Asides sodium, land application of ADE increases the overall risk of overloading arable land with metals—including heavy metals—and non-metals (Golovko et al., 2022; Baldasso et al., 2023; Wang et al., 2023). This increases the risk of heavy metal leaching into groundwater and the transfer of heavy metals to humans and/or animals through food crops.

There are growing efforts therefore, to develop cost-effective and environmentally benign approaches for ADE management (Campos et al., 2019; Ujor et al., 2020; Kumar et al., 2022; Okonkwo et al., 2023). With >90% water content, a sustainable ADE management approach should involve water recovery. Increasing impacts of climate change, particularly, mounting pressure on fresh water resources call for deft measures to recover water from water-replete wastes—such as ADE—other than municipal sewage in the future. Although different methods have been investigated for ADE treatment, cost remains a major impediment to real-world application of such methods (Campos et al., 2019; Herbes et al., 2020). In this study, we investigated two approaches for nutrient removal from the liquid fraction of ADE. In the first approach, we assessed supplementation of the liquid fraction of ADE as a source of minerals during fumarate production by *R*. *delemar* DSM 905 (hereafter referred to as *R. delemar*). *R. delemar* was chosen for this study because of the ability of *Rhizopus* species to thrive on diverse substrates and environments (Shimada et al., 1983; Pranamuda et al., 1995; Mousavi et al., 2023). Additionally, *R. delemar* produces fumaric acid, an important industrial chemical building block with numerous applications in synthesizing pharmaceuticals, resins and polyamides, and more recently, as a supplement in cattle feed, that leads to ~70% reduction in methane emission (Das and Brar, 2014; Jiménez-Quero et al., 2016; Karaffa et al., 2021). Therefore, we rationalized that cultivation of *R. delemar* in ADE-based medium would reduce the ADE nutrient load, particularly ammonium (NH_4_^+^), total nitrogen (N), phosphate (PO_4_-P), total phosphorus (P), sulfur (S), nitrate (NO_3_-N), copper (Cu), chromium (Cr), and lead (Pb). In the second approach, a culture of phosphate-accumulating organisms (PAOs) was adapted to high phosphate concentrations and then, grown in PO_4_-P - and NH_4_^+^-replete ADE, as a means for reducing the PO_4_-P and NH_4_^+^ concentrations of the ADE.

## 2 Materials and methods

### 2.1 Fumarate fermentation coupled to nutrient sequestration from ADE

**Figure 1:**
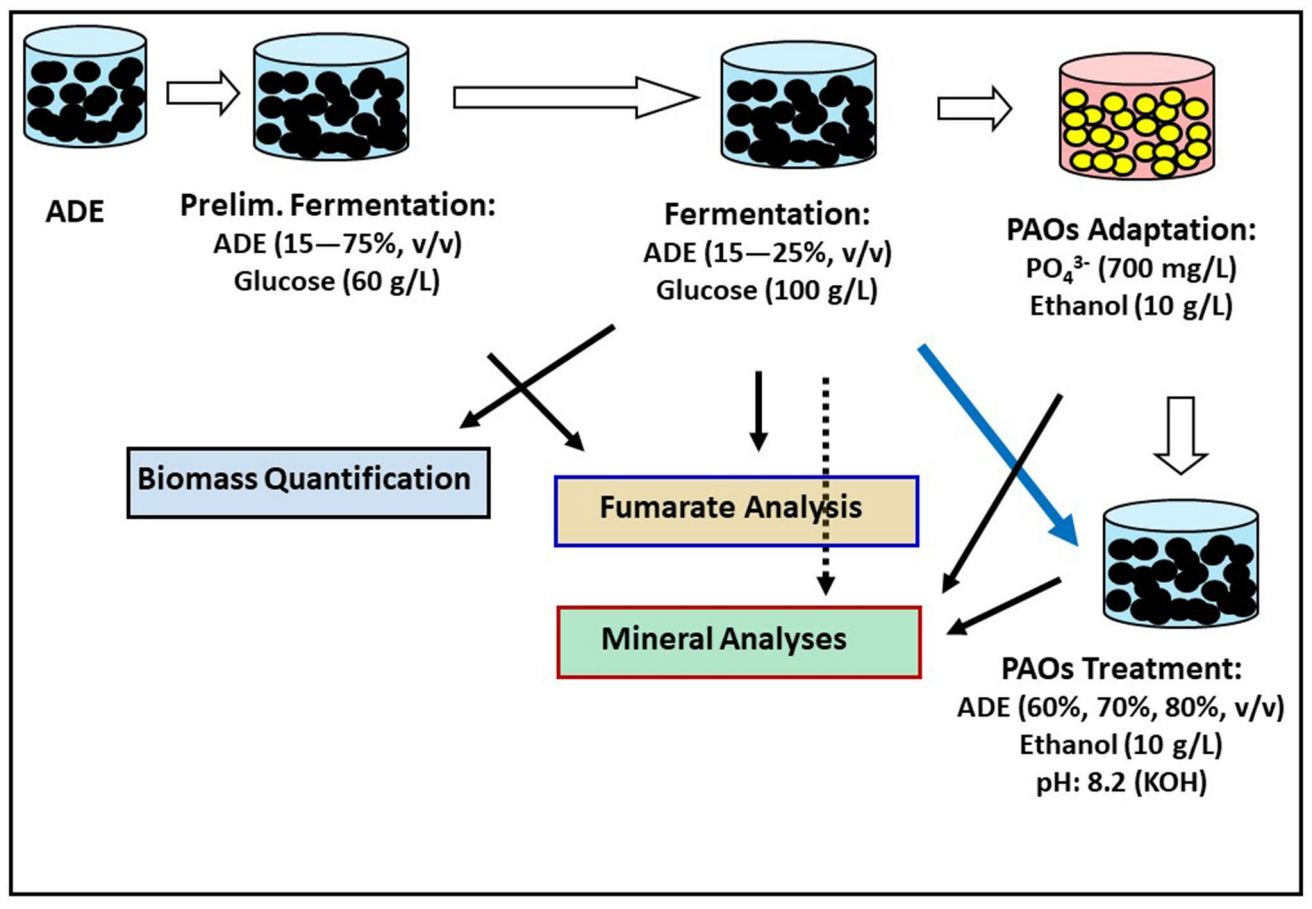
Flowchart of various treatments deployed to assess fumarate production and nutrient removal from ADE-supplemented cultures.

*R*. *delemar* was procured from the German culture collection (Leibniz Institute DSMZ-German Collection of Microorganisms and Cell Cultures, Braunschweig-Süd, Germany). The City of Madison, Wisconsin (United States) community AD facility kindly provided the ADE used in this study. A flowchart of the various treatments adopted for fumarate production in ADE-supplemented cultures are presented in Figure 1. The liquid fraction of the ADE was obtained by centrifuging the ADE five times at 3,000 × g for 30 min in a Jouan CR 312 centrifuge (Thermo Scientific, Waltham, MA, United States). Afterwards, the resulting liquid fraction of the ADE was incubated at 80°C for 12 h in a Thermo Scientific Heratherm incubator oven (Waltham, MA, United States) to eliminate residual microbial cells. Sterilization at higher temperatures led to precipitation of the ADE constituents. Different fermentation media were formulated to initially determine the ideal rate of ADE supplementation. Subsequently, the predetermined ADE supplementation rates were used in batch fermentation. To determine the optimum ADE supplementation rate in the fumarate fermentation medium, a preliminary experiment was set up with different concentrations of ADE. *R. delemar* was grown in media containing a final glucose concentration of 60 g/L supplemented with 25, 50, and 75% (v/v) ADE liquid fraction without buffering. The media were aseptically inoculated with mycelial suspension prepared by cutting mycelial-agar plugs from the front of actively spreading mycelia on potato dextrose agar. Approximately 10 mycelial-agar plugs excised with sterile scalpel blade were placed in sterile 50 mL tubes containing 12 mL of sterile distilled water and approximately 2.5 g of sterile glass beads (Fisher Scientific, Waltham MA, United States). The tubes were shaken vigorously for 2–3 min to shred the mycelia mat into a suspension. Afterwards, 100 mL of each medium above was inoculated with 2 mL of mycelial suspension in sterile 250 mL Erlenmeyer flasks plugged with sterile foam bung. The cultures were incubated for 84 h in a MaxQ 4,450 shaker incubator (Fisher Scientific, Waltham MA, United States) at 150 rpm and 30°C. The cultures were not buffered to determine if the ADE provided a degree of buffering. Non-buffered cultures containing glucose (60 g/L) with and without minerals [ZnSO_4_.7H_2_O (0.088 g/L), KH_2_PO_4_ (0.6) and MgSO_4_ (0.25)] were inoculated and incubated as the ADE-containing cultures as controls.

*R. delemar* cultures supplemented with 25% (v/v) ADE produced the highest amount of fumarate. Therefore, subsequent fermentations were supplemented with 25% (v/v) ADE, except where stated otherwise. For fumarate fermentation, both the test (ADE-supplemented) and control media contained glucose (60 g/L) and yeast extract (1.0 g/L). Subsequent analysis of the mineral constituents of the batch of ADE used during fumarate fermentation revealed a low zinc concentration, and zinc has been shown to play an important role in the growth of *R. delemar* (Wilson, 2015). Hence, the ADE-containing cultures were supplemented with ZnSO_4_.7H_2_O to the same concentration as the control cultures (0.088 g/L). Additionally, our analysis showed that the ADE medium was low on phosphate. Thus, the ADE-containing medium was supplemented with 0.25 g/L KH_2_PO_4_ as a source of phosphate. The control medium was prepared according the recipe described by Sebastian et al. (2021), which contained (in g/L): glucose (60), yeast extract (1.0), KH_2_PO_4_ (0.6), MgSO_4_ (0.25), and ZnSO_4_.7H_2_O (0.088). Both the control and the test (ADE-containing) media were buffered with CaCO_3_ (6.0 g/L). Fermentation cultures were aseptically inoculated with the mycelial suspension of *R. delemar* as described earlier. The cultures were incubated in a MaxQ 4,450 shaker incubator (Fisher Scientific, Waltham MA, United States) at 150 rpm and 30°C. Samples (1.0 mL) were drawn every 6–12 h, spun at 7,000 × g in a Sorvall Legend Micro 21R centrifuge (Thermo Scientific, Waltham, MA, United States) for 5 min and then, the supernatant was stored at 4°C until future analysis. In an attempt to increase fumarate yield, additional experiment was conducted with 15 and 25% (v/v) ADE and the control medium (without ADE) containing 100 g/L glucose and 30 g/L CaCO_3_.

At the end of fermentation, the cultures were spun down in an Allegra X-30R centrifuge (Beckman-Coulter, Pasadena, CA, United States) at 7,000 × g for 10 min. Subsequently, the supernatants were drained off and the resulting pellets were placed on pre-weighed filter papers, which were then placed in a Thermo Scientific Heratherm incubator oven (Waltham, MA, United States) for 1 week. Afterwards, the filter papers and fungal biomasses were reweighed to estimate the weights of the mycelial pellets. All cultures were grown and analyzed in triplicate.

### 2.2 PAOs-mediated removal of nutrients from ADE

PAOs were assessed for nutrient sequestration from ADE. A culture of PAOs was obtained from the city of Madison, WI, United States, Sewerage District. For the PAOs experiments, fresh PO_4_-P-and NH_4_^+^-replete ADE was obtained from the City of Madison, Wisconsin (United States) community AD facility. The solid fraction of the ADE was removed by centrifugation as described earlier. First, the PAOs were adapted to higher than normal phosphate concentrations by supplementing the culture with incremental concentrations of phosphate using K_2_HPO_4_ (10–700 mg/L). Ethanol (10 g/L) was supplied as carbon source. The cultures were grown in cycles of anaerobic (4 h) and aerobic (5 h) conditions in a MaxQ 4,450 shaker incubator (Fisher Scientific, Waltham MA, USA) at 150 rpm and 25°C. When 700 mg/L phosphate was reached (after 1 week of adaptation to incremental phosphate supply), the adapted culture of PAOs was mixed with ADE at varying concentrations (60, 70, and 80%, v/v ADE). All cultures were supplemented with ethanol (10 g/L) as carbon source. K and Mg ions have been reported to enhance PO_4_-P uptake, by counteracting the triple negative charge of PO_4_-P (Pattarkine and Randall, 1999). In addition, pH in the range of 7.5–8.5 has been reported to enhance biological phosphate removal (Schuler and Jenkins, 2002; Serafim et al., 2002). Therefore, the pH of the ADE-supplemented cultures were adjusted to 8.2 using KOH, after mixing with the PAOs suspension. The KOH doubled as a source of K and as a buffer. The cultures were grown over 2 anaerobic-aerobic cycles as described above (18 h). Subsequently, PO_4_-P, P, NH_4_^+^ and N concentrations were analyzed using inductively coupled plasma spectrometry. Cultures were grown and analyzed in triplicate.

### 2.3 Analytical procedures

Changes in pH during the growth of *R. delemar* and the PAOs were monitored using Orion Star A214 pH meter (Thermo Fisher Scientific, Waltham, MA, United States). Fumarate concentration was quantified by high performance liquid chromatography (HPLC). The analytical system consisted of a Dionex U3000 HPLC (Thermo Scientific, Sunnyvale, CA, United States), equipped with an autosampler, fluorescence and a diode array detector (Dionex Ultimate 3000 Fluorescence detector). The HPLC column was a Rezex ROA-Organic Acid H^+^ column (8%) with a dimension of 300 × 7.8 mm (length and internal diameter, respectively; Phenomenex, Terrance, CA, United States). The mobile phase was 0.005 M H_2_SO_4_ and the flow rate was 1.0 mL/min. The column and detector were maintained at 60°C, the run time was 12 min, and an injection volume of 10 μL was used. Fumarate was detected at 210 nm.

Glucose concentration was measured with a YSI 2900 Series biochemistry analyzer (YSI, Yellow Springs, OH, United States) equipped with glucose calibrator, and configured with YSI 2357 buffer and the YSI 2365 glucose oxidase enzyme membrane, according to the manufacturer’s instruction. Samples were diluted (10x) in 15% saline and filtered through 0.22 μm sterile PTFE filter with 13 mm diameter (CELLTREAT, Pepperell, MA, United States). Glucose analysis was conducted with a sample volume of 25 μL. The mineral constituents of test (supplemented with 25%, v/v ADE) and control media were quantified by inductive coupled plasma optical emission spectroscopy (ICP-OES) using Agilent 5,110 ICP-OES unit (Agilent Technologies Inc., Wilmington, DE, United States) as previously described (Ujor et al., 2020). Ethanol concentrations in the PAOs cultures were monitored by gas chromatography as previously described (Agyeman-Duah et al., 2022).

### 2.4 Statistical analysis

Maximum fumarate, glucose consumed, fumarate yield, fumarate productivity, residual metals, and non-metals in 25% ADE cultures were compared to the control cultures using the General Linear Model in Minitab 21 (Minitab Inc., State College, PA). To determine significant differences between the parameters in the 25% ADE and the control cultures, one-way analysis of variance (ANOVA) was performed followed by Tukey’s pairwise comparisons. The confidence level for the analysis was set at 95%, and treatments with a *p* < 0.05 were considered statistically significant.

## 3 Results

### 3.1 Fumarate production in ADE-supplemented media

**Figure 2:**
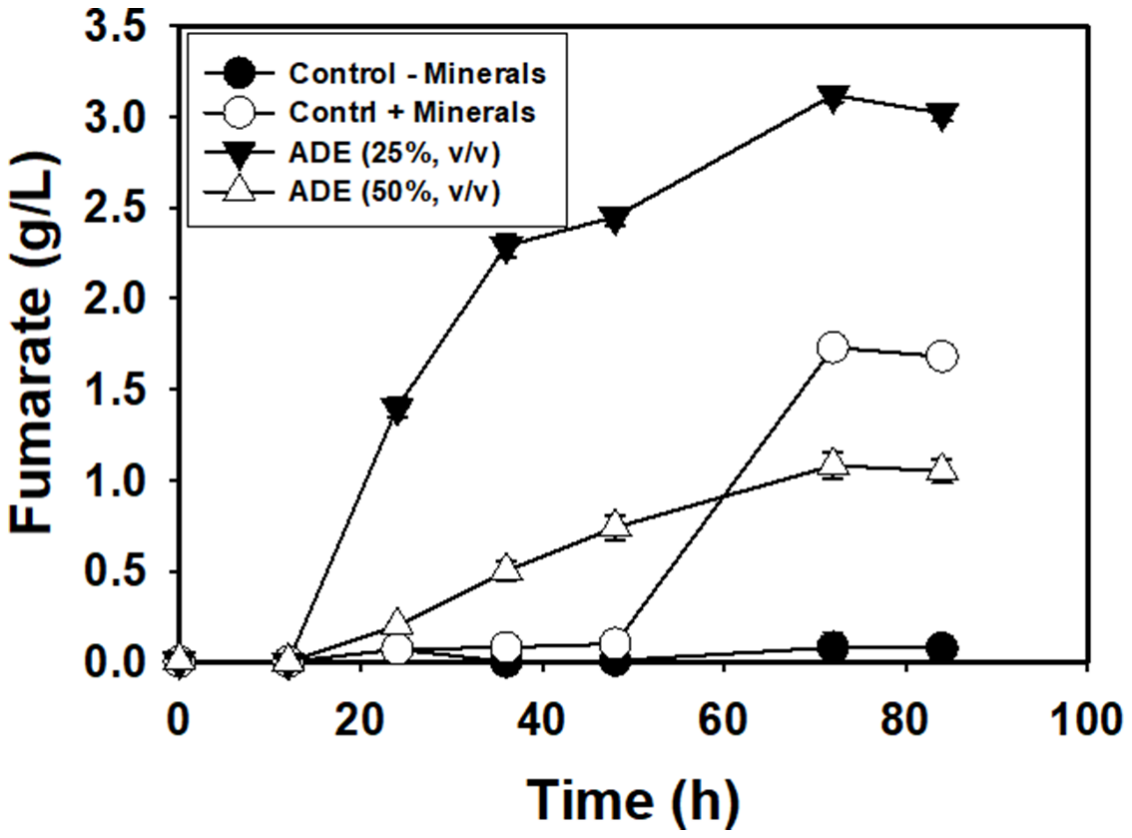
Fumarate concentrations in non-buffered cultures of *R. delemar*.

**Figure 3:**
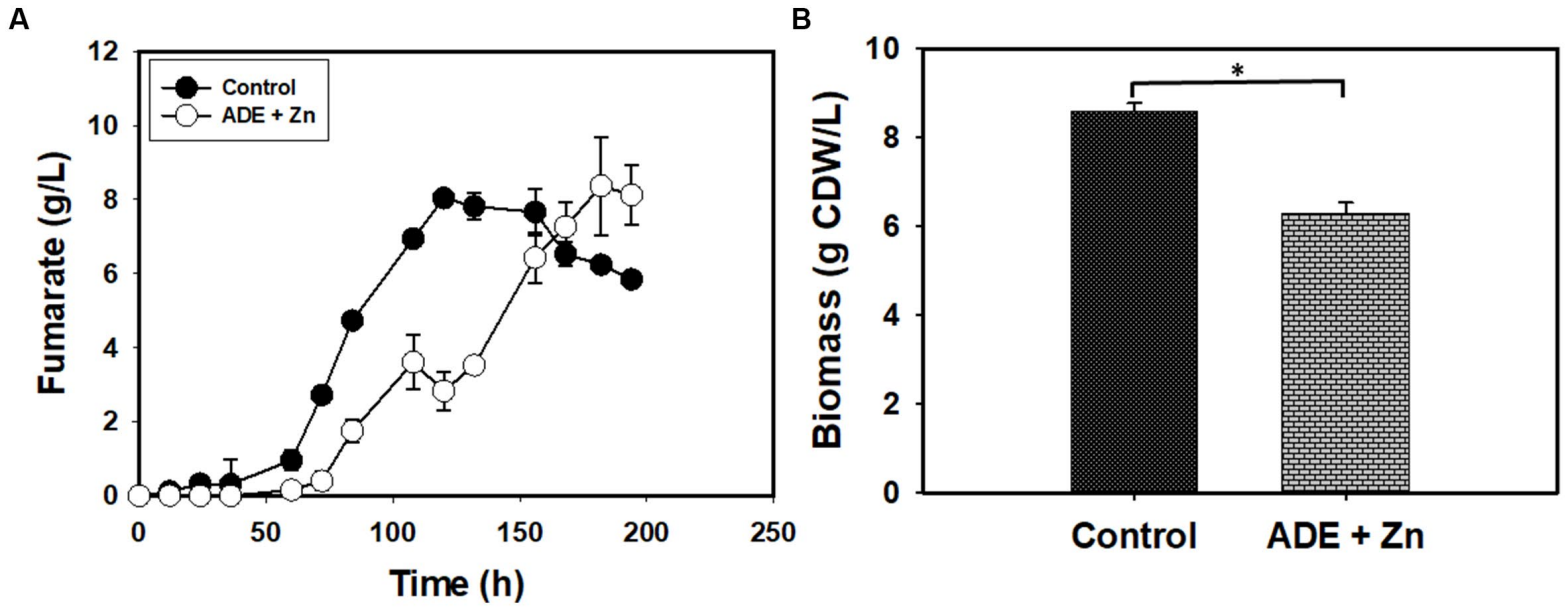
Fumarate and biomass concentrations in CaCO_3_-buffered cultures of *R. delemar* with and without ADE (25%, v/v). **(A)** Fumarate titer. **(B)** Biomass concentration. Asterisk denotes statistical significance relative to the control culture.

**Figure 4:**
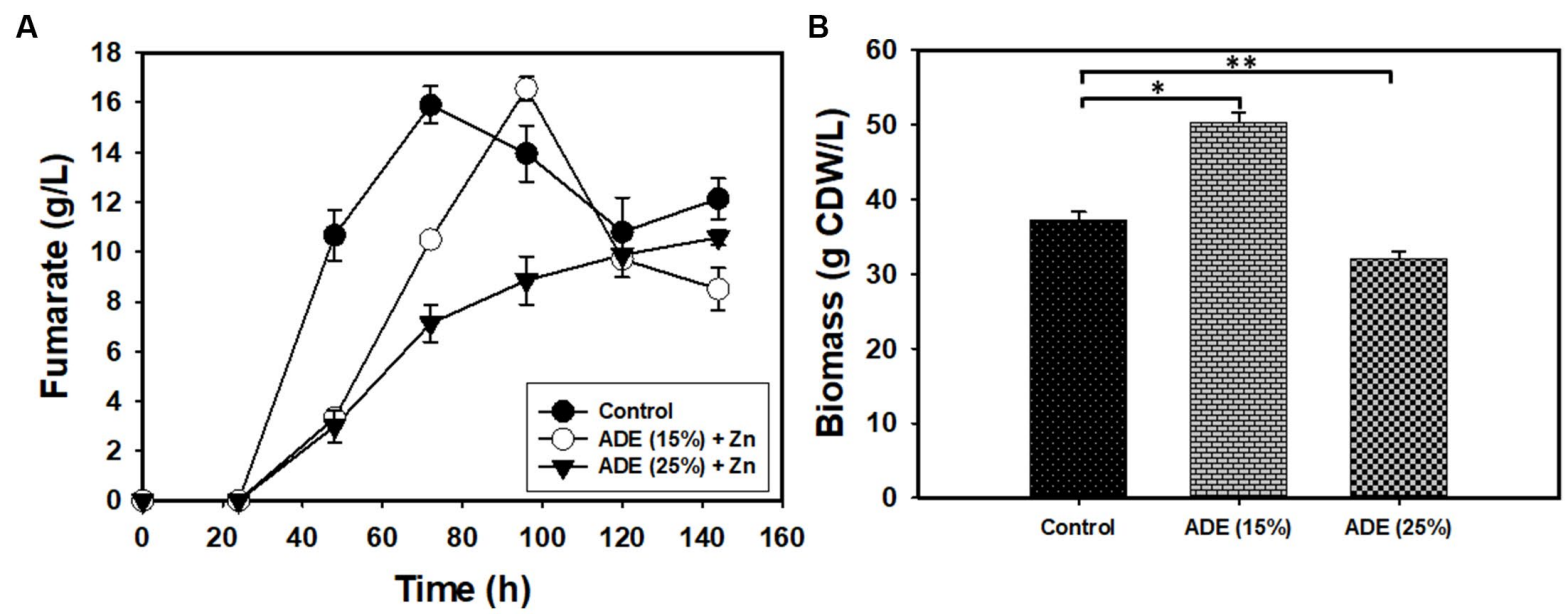
Biomass and fumarate concentrations in cultures of *R. delemar* grown on 100 g/L glucose, with and without ADE (15 and 25%, v/v) supplementation and buffered with 30 g/L CaCO_3_. **(A)** Fumarate concentration. **(B)** Biomass concentration. Asterisks denote statistical significance in comparison to the control medium.

Without buffering, 25% (v/v) ADE produced the highest concentration (3.12 g/L) of fumarate (Figure 2). Non-buffered cultures of *R. delemar* grown in the control medium with mineral supplementation produced 1.73 g/L fumarate—45% less fumarate than the cultures supplemented with 25% (v/v) ADE. In parallel, poor growth and fumarate production were observed in the 50% (Figure 2) and 75% (v/v) ADE (data not shown). With 60 g/L glucose and 6 g/L CaCO_3_, the control medium exhibited greater fumarate productivity (0.07 g/L/h) than the 25% (v/v) ADE (0.05 g/L/h) (Table 1). Nonetheless, the ADE (25%, v/v)-supplemented and un-supplemented cultures of *R. delemar* produced similar maximum concentrations of fumarate (8.4 and 8.04 g/L, respectively) (Figure 3A). Concomitantly, the ADE (25%, v/v)-supplemented cultures produced 27% less biomass than the control cultures (Figure 3B). With 30 g/L CaCO_3_ and 100 g/L glucose, *R. delemar* produced a maximum of ~16 g/L fumarate in 72 h (productivity = 0.22 g/L/h) (Figure 4A) in the control medium. On the other hand, 15 and 25% (v/v) ADE produced ~17 and 11 g/L fumarate at 96 and 144 h, respectively, which translate to respective productivities of 0.17 and 0.07 g/L/h. Notably, increasing glucose and CaCO_3_ concentrations led to ~500% increase in the concentration of *R. delemar* biomass in cultures supplemented with 25% (v/v) ADE (Figures 3B, 4B). However, substantial increase in the biomass concentration of *R. delemar* following increases in the concentrations of glucose and CaCO_3_ led to a 37.5% increase in fumarate titer (Figures 3A, 4A). It is important to note however, that by lowering the ADE concentration from 25 to 15% and increasing glucose and CaCO_3_ concentrations to 100 and 30 g/L, respectively, *R. delemar* biomass concentration increased 41 and 58%, respectively, relative the control and the 25% (v/v) ADE (Figures 3B, 4B). Although growth reduced in the 25% (v/v), when compared to the control and the 15% (v/v), glucose utilization was unaffected. In fact, with 25% ADE, glucose consumption increased 12 and 50% relative to the 15% (v/v) ADE and the control, respectively (Table 1).

**Table 1:** The performance of ADE-supplemented fermentations, relative to the control.

Parameters	Treatments	Media	
		Glucose (60 g/L) + CaCO_3_ (6 g/L) + minerals	Glucose (100 g/L) + CaCO_3_ (30 g/L) + minerals
Glucose consumed (g/L)	Control	57.64 ± 1.04^a^	61.4 ± 2.25^a^
	ADE (25%)	60.00 ± 0.00^b^	92.17 ± 3.15^b^
	ADE (15%)	–	82.40 ± 1.22^c^
Maximum fumarate conc. (g/L)	Control	8.04 ± 0.21^a^	15.90 ± 0.744^a^
	ADE (25%)	8.40 ± 1.32^a^	10.60 ± 0.31^b^
	ADE (15%)	–	16.60 ± 0.47^a^
Fumarate yield (g/g glucose)	Control	0.14 ± 0.07^a^	0.26 ± 0.07^a^
	ADE (25%)	0.14 ± 0.05^a^	0.12 ± 0.06^a^
	ADE (15%)	–	0.20 ± 0.08^a^
Fumarate productivity (g/L/h)	Control	0.07 ± 0.04^a^	0.22 ± 0.04^a^
	ADE (25%)	0.05 ± 0.08^a^	0.07 ± 0.03^b^
	ADE (15%)	–	0.17 ± 0.06^ab^

Tukey’s pairwise comparisons were conducted between treatments (glucose consumed, maximum fumarate concentration, Fumarate yield, and productivity). Treatments with different superscripts within a column are significant at *p* < 0.05.

### 3.2 Sequestration of ADE-borne minerals during fumarate fermentation

**Figure 5:**
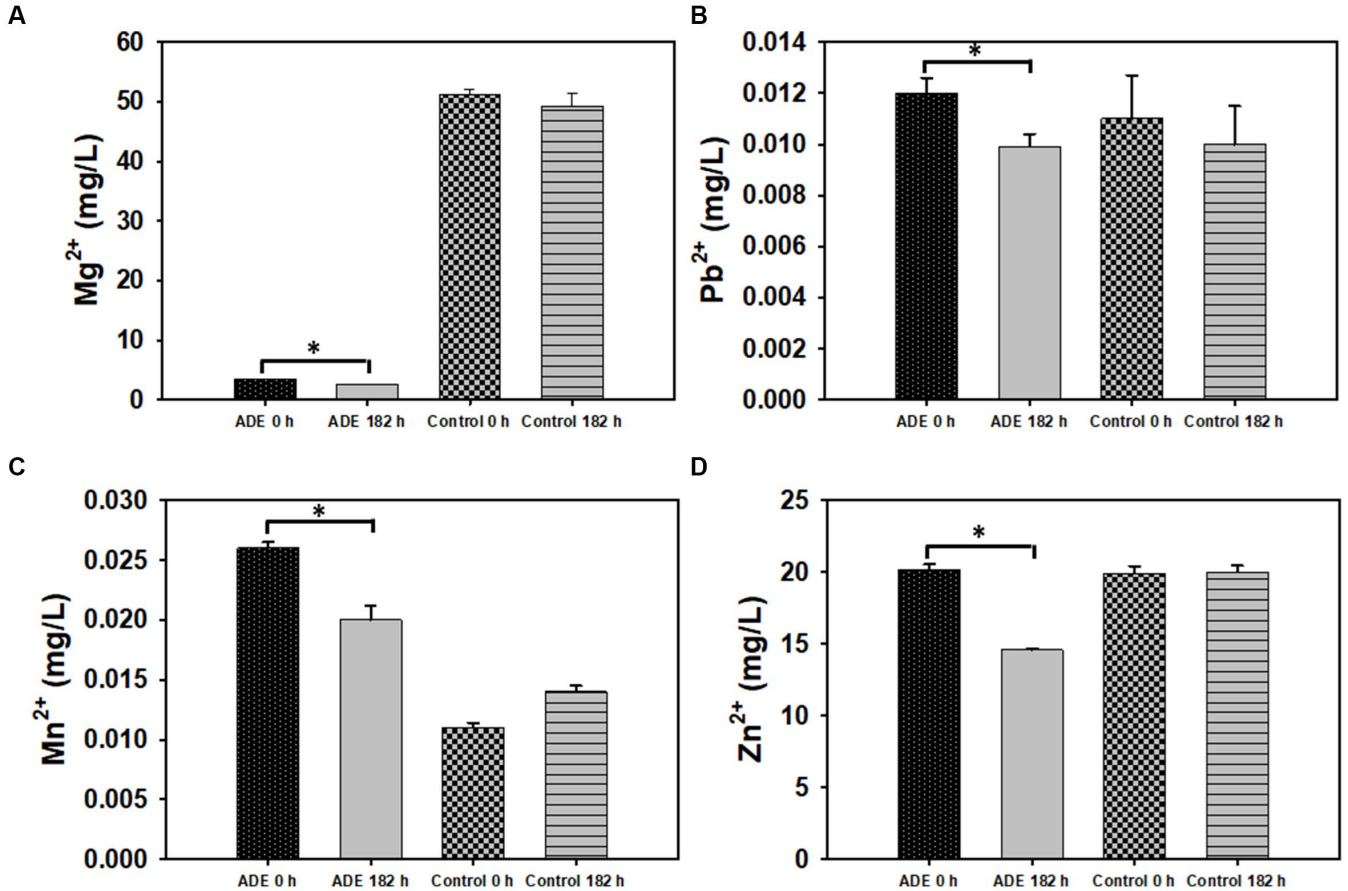
The concentrations of select metals in ADE (25%, v/v)-supplemented and un-supplemented cultures of *R. delemar* before and after fumarate fermentation. **(A)** Magnesium (Mg^2+^) concentration. **(B)** Lead (Pb^2+^) concentration. **(C)** Manganese (Mn^2+^) concentration. **(D)** Zinc (Zn^2+^) concentration. Asterisks denote statistical significance relative to 0 h of fermentation.

A central thrust of this study was to reduce the concentrations of minerals in ADE-supplemented cultures, such that the resulting effluent would be amenable to conventional wastewater treatment technologies. As depicted in Table 2, ADE-supplemented and un-supplemented cultures of *R. delemar* showed similar metal concentrations after fumarate fermentation. Interestingly, addition of ADE (25%, v/v) to the fermentation medium did not result in greater metallic content, relative to the un-supplemented medium (Table 2). The concentrations of As, B, Ba, Be, Ca, Cd, Co, Li, Mo, Na, Ni, Sb, Si, Sr., and V did not undergo significant variations during the growth of *R. delemar* (Table 2), both in the ADE (25%, v/v)-supplemented and un-supplemented cultures. On the other hand, the concentrations of Al, Cr, Cu, Fe, and K decreased in both the ADE (25%, v/v)-supplemented and un-supplemented cultures of *R. delemar*, albeit to varying degrees (Table 2). Notably, the concentrations of Al, K and Cu reduced 40.20, 12.3, and 74.40%, respectively, in the ADE (25%, v/v)-supplemented culture (Table 2). Conversely, the concentrations of the same metals reduced 25.40%, marginally (2.00%) and 43.00%, respectively, in the control cultures. Similarly, the concentrations of Mg, Mn, Pb, and Zn reduced 26.00, 23, 17.50, and 28.00%, respectively, with ADE supplementation (Table 2; Figure 5). On the contrary, statistically, the concentrations of the same metals barely decreased in the control medium (Table 2).

**Table 2:** The concentrations of metals in ADE-supplemented and un-supplemented cultures of *R. delemar* during fumarate fermentation.

Element	Undiluted ADE (mg/L)	25% (v/v) ADE (0 h; mg/L)	25% (v/v) ADE (182 h; mg/L)	% reduction in conc.	Control medium (0 h; mg/L)	Control medium (182 h; mg/L)	% reduction in conc.
Al	0.1006 ± 0.0015	0.082 ± 0.0061^a^	0.049 ± 0.0147^b^	40.20	0.063 ± 0.0056^a^	0.047 ± 0.0042^b^	25.40
As	0.0092 ± 0.0006	<0.004 ± 0.000^a^	<0.004 ± 0.000^a^	0.00	<0.004 ± 0.0000^a^	<0.004 ± 0.0000^a^	0.00
B	3.253 ± 0.0372	0.942 ± 0.0088^a^	1.02 ± 0.0195^b^	0.00	0.005 ± 0.0004^a^	0.0053 ± 0.0211^a^	0.00
Ba	3.26 ± 0.0351	0.124 ± 0.0126^a^	0.115 ± 0.0021^a^	7.30	0.120 ± 0.0150^a^	0.13 ± 0.0212^a^	0.00
Be	<0.004 ± 0.000	<0.004 ± 0.000^a^	<0.004 ± 0.000^a^	0.00	<0.004 ± 0.000^a^	<0.004 ± 0.000^a^	0.00
Ca	2,544 ± 2.0817	1,419 ± 98.330^a^	1,266 ± 11.15^a^	11.00	1,410 ± 92.81^a^	1,215 ± 79.170^a^	14.00
Cd	<0.004 ± 0.000	<0.004 ± 0.000^a^	<0.004 ± 0.000^a^	0.00	<0.004 ± 0.000^a^	<0.004 ± 0.000^a^	0.00
Co	<0.004 ± 0.000	<0.004 ± 0.000^a^	<0.004 ± 0.000^a^	0.00	<0.004 ± 0.000^a^	<0.004 ± 0.000^a^	0.00
Cr	0.013 ± 0.0015	0.0048 ± 0.0002^a^	0.0040 ± 0.0004^b^	17.20	0.008 ± 0.0004^a^	0.006 ± 0.0006^b^	25.00
Cu	0.63 ± 0.0069	0.18 ± 0.0018^a^	0.046 ± 0.0053^b^	74.40	0.014 ± 0.0012^a^	0.008 ± 0.0022^b^	43.00
Fe	0.162 ± 0.0021	0.11 ± 0.0057^a^	0.004 ± 0.0013^b^	96.40	0.079 ± 0.0116^a^	0.0059 ± 0.0012^b^	93.00
K	401.4 ± 4.543	210.2 ± 3.8940^a^	184.1 ± 10.0858^b^	12.30	246.0 ± 8.2585^a^	241.2 ± 39.8245^a^	2.00
Li	0.211 ± 0.0248	0.06 ± 0.0206^a^	0.06 ± 0.0018^a^	0.00	0.06 ± 0.0018^a^	0.068 ± 0.0050^a^	0.00
Mg	10.70 ± 0.1156	3.50 ± 0.0275^a^	2.60 ± 0.0588^b^	26.00	51.13 ± 1.0162^a^	49.3 ± 2.0763^a^	3.60
Mn	0.072 ± 0.0009	0.026 ± 0.0005^a^	0.02 ± 0.0012^b^	23.10	0.011 ± 0.0004^a^	0.014 ± 0.0005^b^	0.00
Mo	0.0058 ± 0.000	<0.0007 ± 0.000^a^	<0.0007 ± 0.000^a^	0.00	<0.0007 ± 0.000^a^	<0.0007 ± 0.000^a^	0.00
Na	143.36 ± 1.8017	50.00 ± 0.9352^a^	53.61 ± 0.9938^b^	0.00	2.31 ± 0.1294^a^	7.70 ± 1.5054^b^	0.00
Ni	0.0088 ± 0.0001	0.0029 ± 0.0004^a^	0.003 ± 0.0007^a^	0.00	0.0014 ± 0.0007^a^	0.0009 ± 0.0008^a^	36.00
Pb	0.044 ± 0.0036	0.012 ± 0.0006^a^	0.0099 ± 0.0005^b^	17.50	0.011 ± 0.0017^a^	0.010 ± 0.0015^a^	9.10
Sb	0.017 ± 0.0015	0.0104 ± 0.0021^a^	0.013 ± 0.0024^a^	0.00	0.0097 ± 0.0030^a^	0.012 ± 0.0023^a^	0.00
Si	8.20 ± 0.1821	4.74 ± 0.0517^a^	5.04 ± 0.0773^b^	0.00	0.54 ± 0.0110^a^	0.82 ± 0.1616^b^	0.00
Sr	0.102 ± 0.0009	1.50 ± 0.1620^a^	1.49 ± 0.0136^a^	0.00	1.50 ± 0.1962^a^	1.70 ± 0.2904^a^	0.00
V	0.003 ± 0.0001	0.0021 ± 0.0004^a^	0.0016 ± 0.0002^a^	24.00	0.0014 ± 0.0002^a^	0.0015 ± 2.7E-19^a^	0.00
Zn	0.13 ± 0.0030	20.21 ± 0.3355^a^	14.60 ± 0.1069^b^	28.00	19.94 ± 0.4793^a^	20.00 ± 0.4474^a^	0.00
Total	3,115.531	1,710.817	1,528.767	11.00	1,731.809	1,533.542	11.45

Tukey’s pairwise comparisons were conducted between the concentrations of metals at 0 h and after 182 h of fermentation for 25% (v/v) ADE and control. Treatments with different superscripts across a row are significant at *p* < 0.05.

Fe concentrations before and after fermentation show strong sequestration of Fe from both the ADE-supplemented and un-supplemented media (Table 2). However, it is worth noting that although a similar percentage of Fe was sequestered from both media, due to 28.2% higher Fe concentration in the ADE-supplemented medium, a higher actual concentration of Fe (0.106 mg/L) was removed from this medium than from the control medium (0.073 mg/L). Because CaCO_3_ was added to both sets of cultures as a buffer, calcium concentrations were high and similar in both the control (1,410 mg/L) and ADE-supplemented (1,419 mg/L) cultures (Table 2). Calcium concentrations did not undergo under significant decreases in both the ADE-supplemented and un-supplemented cultures.

**Figure 6:**
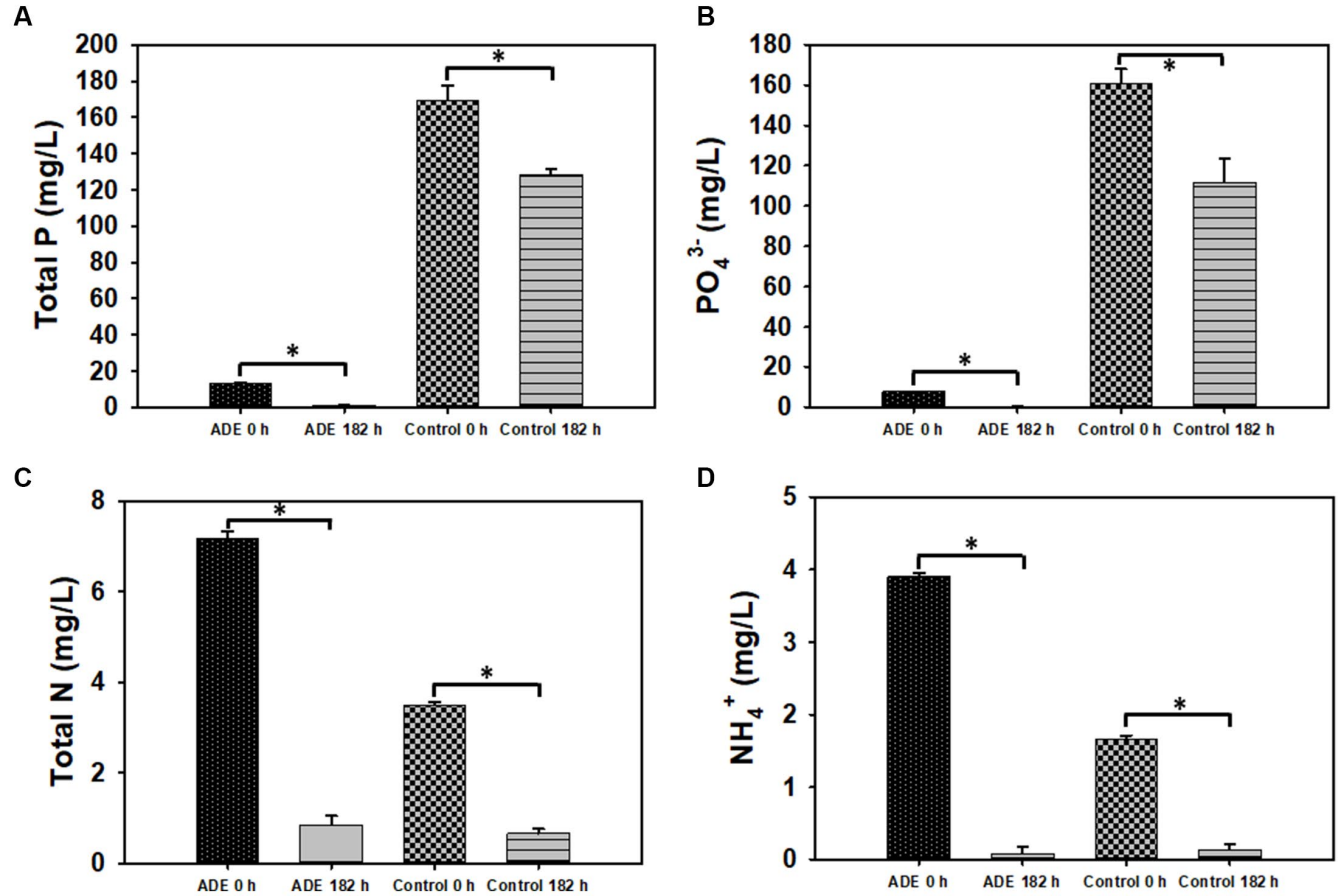
Residual concentrations of select inorganic nutrients following fermentation in ADE (25%v/v)-supplemented and un-supplemented cultures of *R. delemar* before and after fumarate fermentation. **(A)** Total phosphorus concentration (P). **(B)** Phosphate concentration. **(C)** Total nitrogen (N). **(D)** Ammonium concentration. Asterisks denote statistical significance relative to 0 h of fermentation.

As shown in Table 3, the concentrations of non-metals and non-metallic ionic species in both the test and cultures decreased significantly, relative to the degrees of reductions observed for metals. Nitrite (NO_2_-N) and chloride (Cl^−^) were discernibly detected only at 0 h of fermentation but not at 182 h for both the test and the control cultures. Similarly, both ions could not be distinctively quantified in the undiluted ADE. P and PO_4_-P concentrations decreased 93.0 and 97.1%, respectively, in the ADE-supplemented cultures (Table 3; Figure 6). On the hand, the concentrations of both P and PO_4_-P reduced 24.21 and 30.50% in the control cultures. Nonetheless greater concentrations of P and PO_4_-P (41.0 and 49.0 mg/L, respectively) were removed from the control cultures than from the ADE-supplemented cultures (12.22 and 7.48 mg/L, respectively). This is because; the ADE-supplemented cultures contained 92.2 and 95.2% less P and PO_4_-P than the control cultures (Table 3; Figures 6A,B). It is important to note that the concentrations of NH_4_^+^, P and PO_4_-P in the batch of ADE used in the fumarate fermentation experiments were significantly lower than the typical concentrations reported in ADE. This is likely because the ADE was stored in the laboratory at room temperature for several months. As a result, NH_4_^+^ in the ADE (see results below) possibly underwent both volatilization and oxidation [to nitrite (NO_2_-N) and nitrate (NO_3_-N)]. Further, it is likely that continued microbial activity in the unsterilized ADE during storage also led to reduced P and PO_4_-P concentrations. Storage of the ADE at cold temperatures led to precipitation of the mineral constituents.

**Table 3:** The concentrations of non-metals and non-metallic ions in ADE-supplemented and un-supplemented cultures of *R. delemar*.

Element/Ion	Undiluted ADE (mg/L)	25% (v/v) ADE (0 h; mg/L)	25% (v/v) ADE (182 h; mg/L)	% reduction in conc.	Control medium (0 h; mg/L)	Control medium (182 h; mg/L)	% reduction in conc.
Total P	9.68 ± 0.1212	13.17 ± 0.3055^a^	0.95 ± 0.0563^b^	93.00	169.33 ± 8.5049^a^	128.33 ± 3.0551^b^	24.21
Total N	21.80 ± 0.1002	7.18 ± 0.1504^a^	0.85 ± 0.1975^b^	88.02	3.50 ± 0.0600^a^	0.66 ± 0.0995^b^	81.14
S	17.20 ± 0.2020	20.77 ± 0.2196^a^	18.00 ± 0.2563^b^	13.34	85.55 ± 1.8586^a^	85.70 ± 1.9667^a^	0.00
Se	<0.0044 ± 0.0000	0.094 ± 0.0009^a^	0.088 ± 0.0064^a^	9.10	0.086 ± 0.0028^a^	0.083 ± 0.0048^a^	3.61
PO_4_-P	5.32 ± 0.3296	7.70 ± 0.0321^a^	0.22 ± 0.1376^b^	97.14	160.67 ± 7.4087^a^	111.67 ± 11.8462^b^	30.50
NO_3_-N	8.22 ± 0.0493	1.11 ± 0.0289^a^	0.34 ± 0.2158^b^	69.40	0.083 ± 0.0163^a^	0.11 ± 0.0450^a^	0.00
NH_4_-N	8.00 ± 0.6023	3.90 ± 0.0529^a^	0.08 ± 0.0942^b^	98.00	1.66 ± 0.0493^a^	0.14 ± 0.0707^b^	92.0
NO_2_-N	ND	1.52 ± 0.0406^a^	ND^b^	ND	<0.022 ± 0.0000	ND	ND
Cl^−^	ND	33.02 ± 0.2234^a^	ND^b^	ND	6.91 ± 0.1911^a^	ND^b^	ND
Total	70.224	88.464	20.530	76.80	427.811	326.693	24.33

Tukey’s pairwise comparisons were conducted between the concentrations of non-metals at 0 h and after 182 h of fermentation for 25% (v/v) ADE and control. Treatments with different superscripts across a row are significant at *p* < 0.05.

The ADE-supplemented cultures contained 51.5 and 57.4% greater concentrations of total nitrogen (N) and NH_4_^+^, respectively, than the control cultures (Table 3; Figures 6C,D). Although both sets of cultures exhibited similar degrees of reduction for N and NH_4_^+^ in percentage, due to higher initial concentrations in the ADE-supplemented cultures, greater actual concentrations of both nutrients were removed from the ADE-supplemented cultures. Specifically, 6.33 and 3.82 mg/L of N and NH_4_^+^, respectively, were sequestered from the ADE-supplemented cultures, whereas 2.84 and 1.52 mg/L, respectively, were removed from the control cultures. Notably, a greater concentration of NO_3_-N was detected in the ADE-supplemented cultures (1.11 mg/L) than in the control cultures (0.083 mg/L). Whereas 69.4% (1.03 mg/L) of NO_3_-N was removed during fermentation in the ADE-supplemented cultures, the concentration of NO_3_-N did not change during fermentation in the control medium.

Contrasting patterns of change in concentration were observed for sulfur (S). Although the control cultures contained 75.7% more S than the ADE-supplemented fermentations, the concentration of this element did not reduce in the control cultures after fermentation (Table 3). Conversely, out of the 20.80 mg/L S detected in the ADE-supplemented cultures at 0 h, 2.8 mg/L (15.40%) was removed at the end of fermentation. Overall, greater reductions in the concentrations of non-metals and non-metallic ionic species—particularly, P and PO_4_-P—were observed in the ADE-supplemented cultures (~77.00%), relative to the control cultures (24.33%). Comparatively, the ADE-supplemented cultures contained 79.4% less non-metals and non-metallic ionic species than the control cultures (Table 3).

### 3.3 PAOs-mediated sequestration of nutrients from ADE

**Figure 7:**
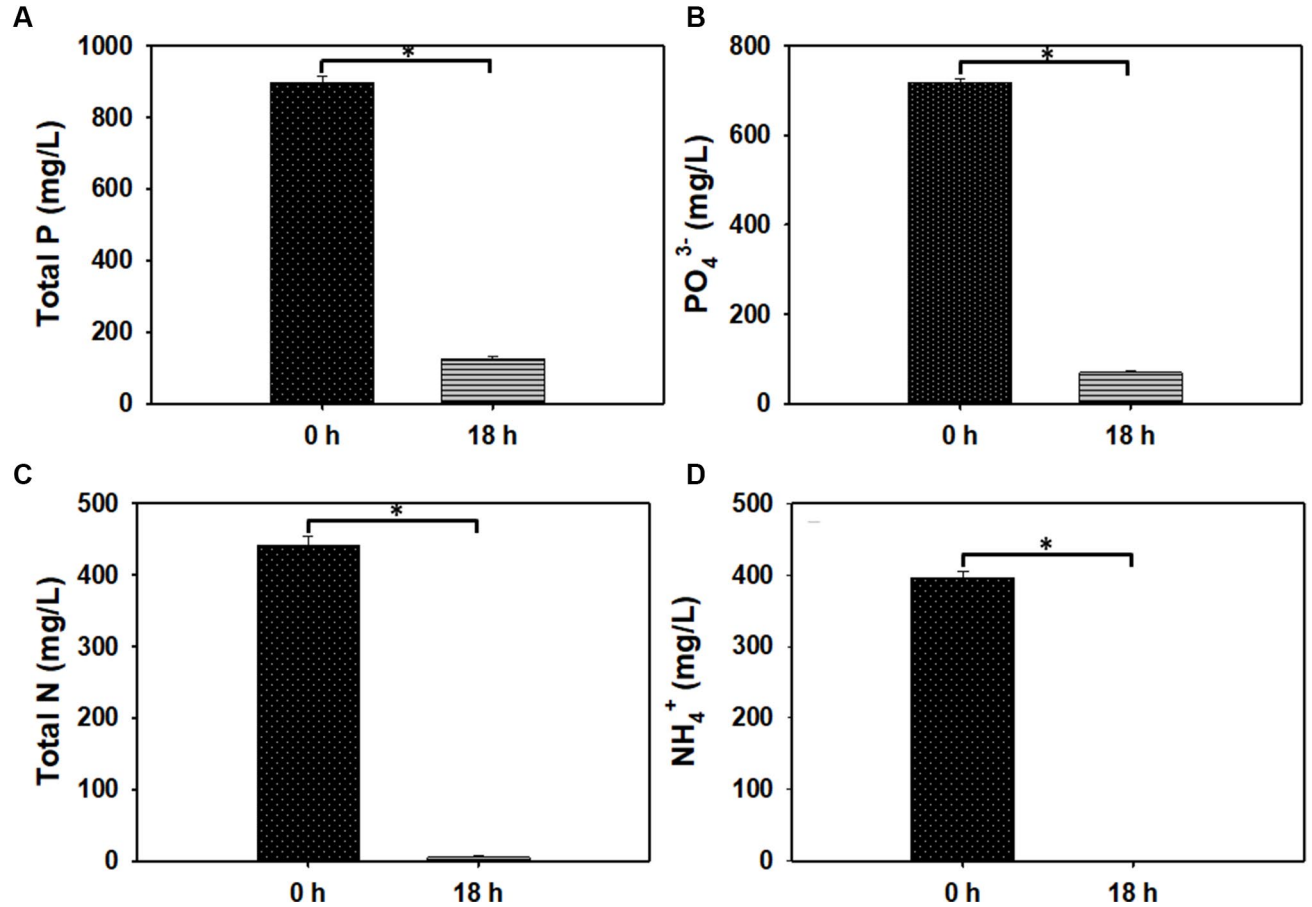
The concentrations of total phosphorus (P), phosphate (PO_4_^3−^), total nitrogen (N), and ammonium (NH_4_^+^) in 60% (v/v) ADE before and after the growth of PAOs. **(A)** P concentration. **(B)** PO43-concentration. **(C)** N concentration. **(D)** NH_4_^+^ concentration. Asterisks denote statistical significance relative to 0 h of fermentation.

Nurmiyanto et al. (2017) showed that excessive PO_4_-P concentration inhibits biological phosphate removal. Thus, in an effort to increase PO_4_-P removal from PO_4_-P-replete ADE, the culture of PAOs was pre-adapted to high PO_4_-P concentration. Following adaptation, cultivation of PAOs in 60, 70, and 80% (v/v) ADE led to varying degrees of reduction in the concentrations of P, PO_4_-P, N, and NH_4_^+^. However, this was more pronounced in the 60% (v/v) ADE. Specifically, in the 60% (v/v) ADE, the concentrations of P, PO_4_-P, N and NH_4_^+^ reduced 86, 90%, ~99, and 100%, respectively (Figure 7). Concomitantly, the ethanol (10 g/L) added to the cultures of PAOs was completely used after 18 h of incubation.

## 4 Discussion

Biological sequestration of ADE-borne nutrients, particularly, PO_4_-P, and NH_4_^+^ has the potential to reduce ADE nutrient loads to levels that may permit economical treatment of the liquid fraction of ADE by existing wastewater treatment technologies. With >90% water content, ADE represents a scarcely researched source of water. Currently, treating ADE is capital intensive relative to municipal sewage, due to high nutrient contents, mainly, PO_4_-P and NH_4_^+^. To determine if biological approaches could be deployed to reduce the nutrient loads of ADE, we evaluated a product (fumarate)-coupled and a non-product coupled approach. A product-coupled approach has the potential to deploy ADE as a source of minerals in biological production of value-added chemicals. Moreover, coupling nutrient removal from ADE to bioproduction of a valuable compound would defray the attendant cost of the sugar used to grow the biological agent—*R. delemar* in this case.

Both the ADE-supplemented and un-supplemented cultures of *R. delemar* produced comparable concentrations of fumarate, irrespective of the sugar and CaCO_3_ concentrations. Apparently, the strain of *R. delemar* used in this study is not a robust producer of fumarate. Nonetheless, the results indicate that ADE exerted some degree of stress on *R. delemar*. Notably, with 15% (v/v) ADE (in medium containing 30 g/L CaCO_3_ and 100 g/L glucose), *R. delemar* produced 70% more fumarate and ~ 42% more biomass than cultures grown in 25% (v/v) ADE (Figure 4B). Additionally, with 6 or 30 g/L CaCO_3_, fumarate productivity in the ADE-supplemented cultures was lower, relative to the control cultures (Figures 3A, 4B). It is likely that a factor present in the ADE negatively affected *R. delemar*. Chemical cleaning agents used in animal facilities are likely to be transferred to the AD process, where animal manure—as in the present study—is a key component of the feedstock. Hence, residual cleaning agents will likely affect fungal/microbial growth during fermentation in ADE-supplemented medium. Hence, careful selection of the source of the ADE is fundamental to potential deployment of this strategy for bioproduction and ADE pretreatment.

Despite the low fumarate yield, the concentrations of Al, Cr, Cu, Fe, K, Mg, Mn, Pb, and Zn reduced significantly (*p* < 0.05) in ADE-supplemented cultures (Table 2). Conversely, the concentrations of K, Mg, Mn, Pb, and Zn, did not undergo significant reductions in the control cultures. Concomitantly, whereas the concentrations of Al, Cu and Fe decreased in the control cultures, stronger decreases were observed in the ADE-supplemented cultures. Apparently, despite containing similar overall metal concentrations, specific metals were more strongly sequestered from the cultures of *R. delemar* supplemented with ADE. Although Al is thought to be largely unconnected to biological processes, Cochrane (1948) suggested that trace amounts of Al alongside Zn, Fe, Cr, and Mn were necessary for improved yield of citric acid by *Aspergillus niger*. While there is no evidence of direct involvement of Al in fumarate production, the concentrations of Al reduced considerably in both the ADE-supplemented and un-supplemented cultures. Thompson and Medve (1984) reported that some ectomycorrhizal fungi were tolerant to Al at 500 mg/L. However, with increasing acidity, Al toxicity increased. Although the Al concentrations in this study were significantly lower than 500 mg/L [0.082 and 0.063 mg/L in the ADE-supplemented and un-supplemented cultures, respectively], acidity amplified considerably with increases in fumarate production, which may likely potentiate Al toxicity, especially in the presence of numerous other metals. This might explain the 40 and 25% reductions in Al concentrations in the ADE-supplemented and un-supplemented cultures, respectively. Stronger sequestration of Al from the ADE-supplemented culture when compared to the control may be ascribed to 30% higher Al concentration in the ADE-supplemented medium, relative to the control. Greater abundance of Al likely triggered increased assimilation, especially if Al-mediated toxicity was prevalent during fumarate production.

Strong Cu removal was observed in both the ADE-supplemented and un-supplemented cultures, albeit more pronounced in the former. Notably, ADE-supplemented cultures contained ~13-fold higher concentration of Cu than the control cultures. We reason that this possibly accounts for the more robust sequestration of Cu from the ADE-supplemented medium. Although Cu is a fungal micronutrient, it is only required at extremely low concentrations, causing severe oxidative damage at relatively low concentrations (Robinson et al., 2021). From an environmental standpoint, robust removal of Cu from the ADE-supplemented medium by *R. delemar* holds some promise for Cu sequestration from ADE.

Due to marginal supplementation of KH_2_PO_4_ and no addition of MgSO_4_ to the ADE-supplemented cultures, the control cultures contained 14.6 and 93.2% greater K and Mg, respectively, than the ADE-supplemented cultures at 0 h (Table 2; Figure 5A). However, the concentrations of both metals reduced 12.3 and 26.0% in the ADE-supplemented cultures, respectively, while barely reducing in the control cultures during fermentation. Apparently, stronger sequestration of K and Mg in the ADE-supplemented cultures cannot be explained by the presence of greater concentrations of these metals—as this was not the case. On the contrary, lower levels of K and Mg in the ADE-supplemented cultures is the likely underlying driving factor for this trend. Both K and Mg are particularly essential nutrients, more so than Al and Cu. This therefore raises the question as to whether the nutritional and metabolic relevance of K and Mg engendered a stronger uptake response to both metals. However, it is important to highlight that residual 210.2 and 2.6 mg/L K and Mg, respectively, remained in the medium post fermentation (Table 2). A form of ‘metabolic urgency’ to reinforce the uptake of K and Mg should have resulted in significantly lower residual concentrations for both metals after fermentation. Thus, the residual concentrations of both metals suggests that metabolic requirements alone do not account for greater K and Mg sequestration from the ADE-supplemented medium. It appears therefore, that other factor(s) might account for the enhanced sequestration of K and Mg in the ADE-supplemented cultures, relative to the control cultures. Interestingly, the control cultures contained 52.0% less Ni than the ADE-supplemented cultures. However, Ni concentration decreased 36.0 and 0.0% in the control and ADE-supplemented cultures, respectively. Ni was the only metal that occurred at a greater concentration in the ADE-supplemented medium but was more strongly sequestered from the control medium (Table 2). The underlying reason for this trend is unclear. However, we speculate that this might be related to cross-uptake of the divalent metals, Mg and Ni. Ni is taken up by the Mg transport system in most organisms, and often, Mg uptake supersedes Ni uptake (Babich and Stotzky, 1983; Joho et al., 1995). Noticeably, as mentioned above, the ADE-supplemented cultures contained 93.2% less Mg than the control cultures. Thus, it is plausible that the lower concentration of Mg—an essential nutrient in most organisms including fungi (Prasad et al., 2019)—favored a stronger Mg uptake over Ni in the ADE-supplemented cultures.

Although both the ADE-supplemented and the control cultures contained identical concentrations of Zn, a 28.0% reduction in Zn concentration was observed in the ADE-supplemented cultures, as opposed to 0.0% in the control cultures. It is not clear as to why both sets of *R. delemar* cultures exhibited contrasting profiles of Zn after fermentation, despite containing identical Zn concentrations at the beginning of fermentation (Table 2). Co-import of Zn, Fe and Cu by the divalent metal transporter 1 (DMT1; Mims and Prchal, 2005) might explain this trend. Whereas both the ADE-supplemented and control cultures contained similar total metal concentrations, the concentrations of specific elements such as Fe, Cu, Na, Ni, and Mn were higher in the ADE-supplemented cultures. Among these metals, Cu, Na, Ni, and Mn exert considerable toxicity on microbial cells (Robinson et al., 2021). Thus, it is likely that *R. delemar* underwent greater metal import in the ADE-supplemented cultures to sequester more toxic metals from the medium, which might account for the drastically lower concentration of Zn—likely due to reduced discrimination among metals imported by DMT1 [encoded by *smf* genes (Smf1p, Smf2p, Smf3p)]—in the ADE-supplemented cultures post fermentation. As did Zn and Cu—both of which are divalent—the concentrations of Pb and Mn reduced ~18 and 23%, respectively, in ADE-supplemented cultures, while no decreases in concentrations were observed for the same metals in the control cultures (Table 2; Figure 5). These results suggest that a mechanism that improves metal uptake, particularly, divalent metals may have been upregulated in the ADE-grown cultures of *R. delemar*, in comparison to the control.

Unsurprisingly, Fe was strongly sequestered from the ADE-supplemented and un-supplemented cultures. Although Fe is typically not considered an environmental threat in waste streams such ADE or landfill leachate, it can indirectly exert far-reaching impacts on aquatic environments. This is because; Fe is a particularly essential nutrient/cofactor in biological cells (Philpott, 2006; Caza and Kronstad, 2013). In fact, Fe is a critical micronutrient for algal growth owing to its vital role in diverse metabolic processes such as photosynthetic electron transport, chlorophyll synthesis, respiration, nitrate reduction, and nitrogen fixation (Wang et al., 2014; Ermis et al., 2020; Liu et al., 2021). Thus, asides the removal of more directly toxic heavy metals, there is considerable merit to Fe removal from waste streams that have the potential to introduce Fe into the environment. Ultimately, this can help to starve algae of a vital nutrient. Similarly, excessive release of K-rich waste into the environment negatively impacts the structural stability of soil, leading to impaired hydraulic conductivity (Arienzo et al., 2009; Liang et al., 2021). Likewise, Cu has been reported to exert toxic effects on aquatic life at 6.3 μg/L (Hall et al., 1998), whereas the ADE used in this study contained 180 μg/L Cu—28.6-fold higher than the toxic threshold. Furthermore, Pb and Mn pose a significant threat to both humans and aquatic organisms (Baker et al., 2015; Wani et al., 2015). Indeed, these results highlight the potential for developing a bio-based approach for nutrient sequestration from ADE, and perhaps, similar nutrient-replete waste streams. It is important however, to mention that CaCO_3_ is an essential buffering agent in fermentative production of fumarate. As a result, this increases the likelihood of introducing high amounts of Ca into the environment post fermentation. Besides increasing soil or water pH, Ca may lead to the mobilization of heavy metals in soil (Fay and Shi, 2012). Therefore, developing a fermentation strategy that limits Ca concentration in the effluent, thereby minimizing the introduction of Ca into the environment is an important research undertaking to consider.

*R. delemar*-mediated nutrient sequestration from ADE-supplemented cultures was not limited to metals. The concentrations of P, PO_4_-P, N, NH_4_^+^, NO_3_-N and S reduced significantly following fumarate fermentation in the ADE-supplemented medium. A considerably higher concentration of NO_3_-N was detected in the ADE-supplemented cultures, when compared to the control. Accordingly, this possibly elicited stronger NO_3_-N removal from the ADE-supplemented medium, relative to the control. Furthermore, the presence of NO_3_-N in the ADE used for fumarate fermentation is indication of NH_4_^+^ oxidation, most plausibly due to prolonged storage of the ADE in the laboratory. Additionally, NO_3_-N has been reported to impinge some toxicity on fungi (Zhou et al., 2017). We speculate that this is likely responsible for the enhanced NO_3_-N removal from the ADE-supplement medium. In fact, the control cultures contained 13.4-fold less NO_3_-N than the ADE-supplemented cultures (Table 3). Hence, if NO_3_-N were physiologically essential to *R. delemar*, the extremely low concentration of NO_3_-N (0.083 mg/L) in the control cultures would have triggered a strong NO_3_-N uptake. On the contrary, NO_3_-N was more strongly sequestered from the ADE-supplemented cultures. In parallel, S concentration showed a greater degree of reduction in the ADE-supplemented cultures, in comparison to the controls. Notably, the control medium contained 4.1-fold greater concentration of S. MgSO_4_ was added to the control medium, but not to the ADE-supplemented medium. We reason that this accounts for the greater amount of S in the control medium. Further, the lack of reduction in the concentration of S in the control cultures suggests that sulfate-might not be a preferred form of S for *R. delemar*. Alternatively, ADE contains residual amino acids, vitamins and nucleic acids, some of which contain S and are typically preferred sources of N and S by most microorganisms (Liu et al., 2009; Möller and Müller, 2012). Hence, greater absorption of such nutrients may explain the higher reduction in concentration observed for S in the ADE-supplemented cultures. With significantly lower concentrations of total S, P, and PO_4_-P, it is plausible that appropriate high-affinity uptake permeases were more strongly expressed during fermentation in the ADE-supplemented medium to scavenge for these nutrients.

Expectedly, NH_4_^+^ was strongly sequestered from both the ADE-supplemented and the control cultures (Table 3). The established role of NH_4_^+^ as an essential biological nutrient underscores the extent of NH_4_^+^ removal from both media. NH_4_^+^ is a key ingredient in most fermentative processes—for bio-production of assorted value-added chemicals—and the industrial Haber-Bosch process, the sole source of NH_4_^+^ in society, is energy intensive, accounting for 1.8% of global energy consumption and 2–3% of global fossil fuel consumption, while generating >560 million tons of CO_2_ (3% of global output)/yr (Smil, 2001; Lehnert et al., 2016; The Royal Society, 2020; Dong et al., 2021; Lehnert et al., 2021). Therefore, given the current scale of industrial production of bio-chemicals and the expected growth in this sector, on account of growing efforts to decarbonize the economy, transitioning to a biobased economy will have far-reaching environmental impacts, unless more sustainable sources of NH_4_^+^ are identified and harnessed. To this end, fresh NH_4_^+^,-rich ADE might be a potential candidate for supplanting or at least, minimizing the demand for synthetic NH_4_^+^ in appropriate biomanufacturing processes. Similarly, with increasing concerns over reducing PO_4_-P reserves (Desmidt et al., 2015), indirect “mining” of PO_4_-P-rich wastes such as ADE by tethering ADE-borne phosphate to fermentative production of bio-chemicals holds considerable promise for enhancing the long-term sustainability of biomanufacturing. For this approach to be successful, however, it is critical to identify/develop microbial strains capable of efficient growth and target compound production in ADE or ADE-supplemented media.

In addition to coupling nutrient sequestration to bioproduction, we assessed the potential of using PAOs to remove P, PO_4_-P, N and NH_4_^+^ from ADE. For this, 60% (v/v) ADE produced the most promising results. More importantly, we demonstrate that prior adaptation of PAOs to higher PO_4_-P can improve the capacity for PO_4_-P sequestration from a PO_4_-P-rich waste stream. Our analysis showed that ethanol (10 g/L) was completely used by the PAOs, with concomitant removal of high amounts of P, PO_4_-P, N and NH_4_^+^ from the ADE. Notably, nutrient removal from higher ADE concentrations (70 and 80%) was not as efficient, albeit promising (data not shown). It is likely that increasing nutrient concentrations with increase in ADE concentration reduced nutrient sequestration. Prior exposure of the PAOs to a maximum of 700 mg/L PO_4_-P may account for the results obtained with 60% (v/v) ADE. This is because; the 60% (v/v) ADE contained ~700 mg/L PO_4_-P. Therefore, adaptation to a greater concentration of PO_4_-P may prove instructive. It is important to note that despite the technical merits of PAOs-mediated nutrient removal from ADE, in reality, the utility of this approach in a real world setting may prove challenging due to the inherent cost. Thus, it may be useful to combine a product-coupled approach with PAOs-mediated nutrient sequestration. Previously, we reported significant removal of a broad range of ADE-borne nutrients following fermentative cultivation of *Saccharomyces cerevisiae* in an ADE-based medium (Ujor et al., 2020). This led to significant increase in ethanol production, although with a considerably high residual PO_4_-P. Therefore, combining both approaches (i.e., growing PAOs in the ADE effluent after growth and ethanol production by *S. cerevisiae*), will likely help to achieve efficient PO_4_-P removal, preceded by bioproduction of a value-added compound alongside pre-removal of a wide range of ADE-borne nutrients. This would entail leaving behind some residual ethanol in the ADE-based medium (after ethanol recovery) as a carbon source to fuel PAOs-mediated nutrient sequestration. We will likely explore this strategy in future studies. The ethanol/other appropriate product resulting from the process would help to defray the attendant operational costs.

## 5 Conclusion

The present study explored the potential of product-coupled and non-product-coupled approaches for the removal of excess nutrients from the liquid fraction of ADE. Our results indicate that both approaches can reduce the ADE nutrient load, particularly, P, PO_4_-P, N, NH_4_^+^, Al, Cu, and Fe. The yield of fumarate by *R. delemar* in this study suggests that a potent producer of fumarate (or another target product) with the robust capacity to sequester nutrients from ADE, may prove efficacious for product-coupled microbial sequestration of excess nutrients from ADE. The PAOs approach proved particularly successful at 60% (v/v) ADE. Ideally, closer to 100% (v/v) ADE will likely be more attractive. Thus, it would be interesting to determine the extent to which PAOs can be adapted, with a view to sequestering greater amounts of nutrients from ADE. In addition, operating the system for a longer period to assess stability would provide considerable insight. Furthermore, tethering product-coupled nutrient sequestration from ADE to post bioproduction nutrient removal by PAOs looks particularly attractive for robust nutrient removal, while making a value-added compound. With growing stress on water resources due to climate change, such radical measures may prove essential in the future.

## Data availability statement

The original contributions presented in the study are included in the article/supplementary material, further inquiries can be directed to the corresponding author.

## Author contributions

EA-D: Investigation, Methodology, Writing – review & editing. CO: Formal analysis, Writing – review & editing. VU: Conceptualization, Funding acquisition, Project administration, Resources, Supervision, Writing – original draft, Writing – review & editing.
